# Insanity and Mental Disorder

**Published:** 1907-11-16

**Authors:** Charles Mercier

**Affiliations:** Physician for Mental Diseases to Charing Cross Hospital.


### MENTAL DISEASES.

### INSANITY AND MENTAL DISORDER.

By CHARLES MERCIER, M.D., F.R.C.P., Physician for Mental Diseases to Charing Cross Hospital.

Insanity and mental disorder are usually regarded as convertible terms, so that if a person is insane he is mentally disordered; and if he is mentally disordered he is insane. The first proposition is, no doubt, always true, though there are cases of insanity in which the mental disorder is so subtle and so difficult to identify that we may be puzzled for a long time in determining of what it consists. But the second proposition is by no means universally true, and a large proportion of the disorders that the alienist is called upon to treat are mental disorders indeed, but are far removed from insanity, and the sufferers from them may never come within measurable distance of being insane. It is very important to bear this distinction in mind, for the sufferers from sane mental disorder are often in dread of becoming insane, and we may be able to reassure them with complete confidence; while the insane, whose mental disorder is difficult to identify and classify, are often regarded and treated as sane, and suffer unjustly. Not only are there many magistrates who refuse to make detention orders with respect to such insane persons, but there are many medical men who hesitate, or even refuse, to sign certificates that such persons are insane, and more who, while recognising that it would be right to make a certificate, are yet puzzled to know in what terms the certificate should be expressed.

This difficulty would disappear if it were recognised and borne in mind that a certificate of lunacy need not specify any disorder of mind at all. The statement seems startling, but it is quite true. What we are called upon to specify are "facts indicating insanity," and if, as I maintain, insanity is not identical with mental disorder, and contains much besides mental disorder, then we can specify "facts indicating insanity" without referring to mental disorder at all. Let me give an instance. I was asked to see and examine a lady with respect to her sanity, and, if I found her insane, to sign a certificate to that effect. I was introduced to her, and said, "How do you do, Mrs. Brown?" She did not utter a word: she jumped up and spat in my face. I made no effort to discover the state of her mind, but without another word went away and wrote a certificate that she was insane; and as a "fact indicating insanity" I described my interview with her. The fact was accepted by the authorities as indicating insanity, and will, I think, be accepted by my readers; but it will be observed that it is not a proof of mental disorder. I do not say that in this case there would have been any difficulty in identifying mental disorder if it had been necessary to do so, but the case is a good illustration of the needlessness of referring to mental disorder, if we can establish insanity, as we often can, without any such reference. There is an impression still lingering in the minds of many medical practitioners that a certificate of insanity must set forth some delusion entertained by the patient. The foregoing case proves not only that this is not necessary, but that it is not even necessary to refer to the mind at all.

The converse case, in which mental disorder exists without any trace of insanity, is very frequent indeed, and forms a large proportion of the cases that seek the advice of the practitioner in mental disorders. The most frequent instance of such disorder, and one that presents itself several times a week, is that of simple melancholy. The patient is depressed in spirits, miserable, wretched, not only without sufficient justification in his circumstances for the depression, but against his own judgment, knowledge and true appreciation of these circumstances. He recognises quite clearly that his depression is out of proportion to the adversity of his circumstances. The majority of these cases of simple melancholy occur in women who lead very lonely lives. Their husbands or breadwinners are away at business from early morning till late evening. They have no children; they have no companionship; they have insufficient occupation; they suffer from the starvation of human companionship and human sympathy and human interests that are inevitable in such circumstances, and they become melancholy. Such is the history of scores of cases; and, when the obvious remedy is applied, they recover without ever having been within measurable distance of insanity.

Simple melancholy, while it is the most frequent case of mental disorder without insanity, yet forms but a small proportion of such cases that apply for treatment, so numerous and so diverse are they. Among children we see the preternaturally dull and the preternaturally clever, the thievish, the cruel, and the erotic. Among adolescents we see those whose diffidence and bashfulness, whose timidity and ready blushes interfere with wholesome association with their fellows, interfere even with earning their livelihood; and we see the more common case of the youngster whose arrogant assertion of his own genius disgusts his associates. We see the whole gamut of morbid sexuality in both sexes, from the girl whose eroticism appals her parents and disgusts her fellows, to the girl who becomes infatuated with a groom or a travelling tinker; from the man whose real or imagined impotence poisons his life and absorbs his attention, to him who wallows in unmentionable orgies, and seeks, in a certificate of lunacy, a handy refuge from the police. We see prodigals of every kind and degree, the facile and the obstinate, the weak-minded and the brilliant, the honest and the dishonest. We see the whisperers, who utter, *sotto voce*, their most secret thoughts, their criticisms on their interlocutors; the post-touchers; the counters; the hesitators; and the army of those who suffer from phobias and manias of many kinds. All these and many others are persons of disordered mind. None of them is insane. Most of them require treatment, and most of them find in treatment an effectual relief; but there are very few of them whom it would not be an outrage to send to a lunatic asylum.

